# Supplementary material for: Hypertension in the United States Fire Service
Source: Int J Environ Res Public Health. 2021 May 19;18(10):5432. doi: 10.3390/ijerph18105432 (PMC8160987; doi:10.3390/ijerph18105432)
Supplement: Supplementary file 1 [file ijerph-18-05432-s001.zip › ijerph-1213121-supplementary.pdf]

International Journal of
Environmental Research and Public Health

MDPI

Table S1. Descriptive statistics of firefighter cohorts across geographical locations.

Characteristics	M-A Cohort Male	M-W Cohort Male	S-W Cohort Male	S-E Cohort Male	M-A Cohort Female	M-W Cohort Female
N	858	3310	558	337	102	172
Age (years)	40.5 ± 9.0	42.5 ± 9.1[a]	42.9 ± 8.9[a]	37.8 ± 10.2[abc]	39.1 ± 8.7	40.0 ± 8.9
White *n* (%)	615 (72)	2965 (91)[a]	445 (83)[ab]	n/a	80 (78)	151 (90)[a]
African American/Black *n* (%)	127 (15)	210 (6)[a]	11 (2)[ab]	n/a	14 (14)	11 (7)[a]
Other *n* (%)	116 (14)	92 (3)[a]	80 (15)[ab]	n/a	8 (8)	5 (3)[a]
Height (m)	1.8 ± 0.1	1.8 ± 0.1	1.8 ± 0.1[ab]	1.8 ± 0.1[ab]	1.7 ± 0.1	1.7 ± 0.1
Body Weight (kg)	92.4 ± 14.3	97.7 ± 17.2[a]	93.1 ± 15.1[b]	94.2 ± 16.3[b]	73.0 ± 12.3	80.3 ± 18.3[a]
Body Mass Index (kg·m^{-2})	28.9 ± 3.9	30.3 ± 4.7[a]	28.7 ± 4.0[b]	28.9 ± 4.4[b]	26.1 ± 4.0	28.7 ± 5.7[a]
Normal *n* (%)[1]	118 (14)	336 (10)[a]	87 (16)[b]	57 (17)[b]	43 (43)	50 (29)[a]
Overweight *n* (%)[2]	430 (51)	1385 (42)[a]	282 (53)[b]	168 (50)[b]	42 (42)	59 (35)[a]
Obese *n* (%)[3]	293 (35)	1575 (48)[a]	166 (31)[b]	112 (33)[b]	14 (14)	61 (36)[a]
Systolic Blood Pressure (mmHg)	120.4 ± 10.6	126.2 ± 10.0[a]	121.8 ±11.8[ab]	121.7 ± 7.5[ab]	114.2 ± 12.6	119.7 ± 10.6[a]
Diastolic Blood Pressure (mmHg)	77.3 ± 7.9	82.9 ± 5.9[a]	78.8 ± 7.2[ab]	77.2 ± 5.3[bc]	72.3 ± 7.7	78.5 ± 7.2[a]
Hypertension n (%)[4]	437 (51)	2541 (78)[a]	322 (60)[ab]	n/a	25 (25)	70 (42)[a]
Antihypertensive Medication n (%)	135 (16)	596 (18)	73 (14)[b]	n/a	6 (6)	25 (15)[a]

Data presented as *n* (%) or mean ± standard deviation. [1] Normal BMI defined as <25 kg·m^{-2}; [2] Overweight BMI defined as 25-29.9 kg·m^{-2}; [3] Obese BMI defined as ≥30 kg·m^{-2}; [4] Hypertension defined as systolic blood pressure ≥130 mmHg, or diastolic blood pressure ≥80 mmHg, or taking antihypertensive medication; M-A, Mid-Atlantic; M-W, Midwest; S-W, Southwest; S-E, Southeast. For males, ANOVA models with *post-hoc* comparisons and chi-squared tests were conducted to test for statistical differences between the cohorts. For females, t-tests and chi-squared tests were conducted to test for statistical differences between the cohorts. [a] Indicates a statistical difference with the M-A Cohort., [b] indicates a statistical difference with the M-W Cohort, [c] indicates a statistical difference with the S-W Cohort. The level of significance for all analyses was considered at $p < 0.05$.
